# Direct differentiation of insulin-producing cells from human urine-derived stem cells

**DOI:** 10.7150/ijms.36011

**Published:** 2019-11-15

**Authors:** Yongha Hwang, Seon-Heui Cha, Yeonhee Hong, Ae Ryang Jung, Hee-Sook Jun

**Affiliations:** 1 College of Pharmacy and Gachon Institute of Pharmaceutical Science, Gachon University, Incheon, 21999, Republic of Korea; 2 Department of Marine Bioindustry, Hanseo University, Chungcheongman-do, 31962, Republic of Korea; 3 Lee Gil Ya Cancer and Diabetes Institute, Gachon University, Incheon, 21936, Republic of Korea; 4 Gachon Medical Research Institute, Gil Hospital, Incheon, 21999, Republic of Korea

**Keywords:** urine-derived stem cell, diabetes, differentiation of insulin producing cell, pancreatic beta-cell

## Abstract

The loss of pancreatic β-cells is a cause of diabetes. Therefore, replacement of pancreatic β-cells is a logical strategy for the treatment of diabetes, and the generation of insulin-producing cells (IPCs) from stem cells has been widely investigated as an alternative source for pancreatic β-cells. Here, we isolated stem cells from human urine and investigated their differentiation potential into IPCs. We checked the expression of surface stem cell markers and stem cell transcription factors, and found that the isolated human urine-derived stem cells (hUDSCs) expressed the stem cell markers CD44, CD90, CD105 and stage-specific embryonic antigen (SSEA)-4. In addition, these cells expressed octamer binding transcription factor (Oct)4 and vimentin. hUDSCs could differentiate into adipocytes and osteocytes, as evidenced by Oil-red O staining and Alizarin Red S-staining of differentiated cells, respectively. When we directly differentiated hUDSCs into IPCs, the differentiated cells expressed mRNA for pancreatic transcription factors such as neurogenin (Ngn)3 and pancreatic and duodenal homeobox (Pdx)1. Differentiated IPCs expressed insulin and glucagon mRNA and protein, and these IPCs also secreted insulin in response to glucose stimulation. In conclusion, we found that hUDSCs can be directly differentiated into IPCs, which secrete insulin in response to glucose.

## Introduction

Human stem cells have been of growing interest for *in vitro* models, disease modeling, and cell-based therapy. As there are significant differences between animal and human physiology, a translation to human biology is sometimes inadequate. Therefore, human stem cells are attractive for better models of human disease and drug reactions than animal models. In addition, stem cells have potential for tissue repair and regeneration.

Diabetes mellitus (DM) is a chronic metabolic disorder characterized by a relative or absolute deficiency in circulating insulin levels, resulting in high blood sugar levels over a prolonged period 1-3. Pancreatic β-cell loss is known to be a major cause of both type 1 and type 2 diabetes 4. Therefore, restoration of insulin-producing β-cells would be a logical strategy for the treatment of both forms of diabetes. Insulin therapy has been used to treat diabetes, but it can have side effects such as hypoglycemic episodes. Islet transplantation is an effective therapy for treating severe type 1 diabetes 5, but there are limitations due to a lack of donors of islet sources 6. In this regard, differentiated insulin-producing cells (IPCs) using human stem cells are an attractive source of alternative pancreatic islets for transplantation 7. In addition, a consistent source of differentiated β-cells from human stem cells would provide a unique and valuable drug discovery platform for diabetes, and could also replace for *in vitro* or *in vivo* model systems 8, 9.

Human urine-derived stem cells (hUDSCs) have advantages, including the easy and inexpensive isolation procedure 10. They can also differentiate into various types of cells, such as adipocytes, osteocytes, chondrocytes, myocytes, and neurons 11-13. However, the differentiation of hUDSCs into IPCs has not been investigated. Therefore, in this study, we isolated hUDSCs according to a previous report, and their potential for use as stem cells was confirmed in accordance with existing reports 12, 14. Finally, we confirmed that isolated hUDSCs are capable of differentiating into insulin-producing β-cells and suitable for use in the treatment of diabetes.

## Materials and Methods

### Ethical approval

The protocol for the collection of urine samples from healthy donors and the isolation of stem cells from urine was reviewed and approved by the Public Institutional Review Board designated by the Ministry of Health and Welfare, Korea (P01-201507-31-002).

### Isolation of stem cells from human urine (hUDSCs)

To isolate stem cells from urine, fresh urine samples (average amount of 50 ml per sample, four subjects) were immediately transferred to the laboratory under sterile conditions at 4°C. Each sample was centrifuged at 500 × g for 5 minutes and the cell pellets were gently resuspended in sterile phosphate buffer saline (PBS) solution. After the cell suspension was centrifuged at 500 × g for 5 minutes, the cell pellets were resuspended in 3 ml of medium composed of 1:1 mixture of DMEM/F12 (ThermoFisher Scientific, MA, USA) and keratinocyte serum free medium (KSFM, ThermoFisher Scientific), containing 5% fetal bovine serum (FBS; ThermoFisher Scientific) and 1% penicillin/streptomycin (Welgene, Deagu, Korea). The cells were seeded (1×104 cells/well) to 24-well plates (Falcon, USA), and incubated at 37°C in a 5% CO2 and air humidified incubator (Heraeus HeraCell 150, Thermo). Culture medium was changed every two days until the cells formed colonies. Each colony was transferred to individual wells of 24-well plate and cultured. The cells were split when they reached 70-80% confluency.

### Cell culture

hUDSCs or human adipose tissue-derived stem cells (hADSCs; Invitrogen, USA) were seeded at 1×10^4^ cells/well in 6-well culture plates. hUDSCs were maintained in DMEM/F12:keratinocyte SFM (1:1, v/v, ThermoFisher Scientific) medium containing 5% FBS (ThermoFisher Scientific), 1% penicillin/streptomycin. hADSC were maintained in MesenPRO medium (Invitrogen) containing supplement, 1% penicillin/streptomycin and 1× GlutaMAX (ThermoFisher Scientific, MA, USA). The cells were incubated in a humidified incubator with 5% CO_2_.

### Flow cytometry

To check the expression of stem cell markers on the surface, the cells at passage 3 were harvested and fixed with 70 % EtOH overnight at 4°C. After washing twice with PBS, cells (5×10^5^ cells) were stained with antibodies against FITC fluorescence-conjugated anti-CD31, anti-CD34, anti-CD44, anti-CD90, anti-CD105, or anti-SSEA-4 antibodies (BD Pharmingen, USA) for 1 h at room temperature. Cells were washed twice with PBS and analyzed on a BD LSR II Flow Cytometer (BD Biosciences, USA).

### Immunocytochemical staining

hUDSCs or hADSCs were cultured on coverslips, washed with PBS and fixed with 4% paraformaldehyde (Biosesang, Korea) for 10 minutes at room temperature. Following fixation, the cells were washed with PBS and then blocked with PBS, containing 1% bovine serum albumin (BSA) and 0.1% Triton X-100, for 30 minutes at room temperature, and incubated overnight with anti-vimentin, anti-E-cadherin, or anti-oct4 (Santa Cruz Biotechnology, Inc., USA) antibodies in a humidified chamber at 4°C. The cells were then incubated with fluorescein-conjugated secondary antibodies (Life technology, USA) and 4',6-diamidino-2-phenylindole (DAPI) (Sigma-Aldrich, MO, USA) for 1 h, in the dark, at room temperature. Following this, the coverslips were mounted with fluorescence mounting medium (DAKO, CA, USA) and cell images were observed under a confocal microscope (Zeiss, Germany).

### Adipogenic and osteogenic differentiation

The cells were seeded (1.5×10^5^ cells/well in 6-well plate) in keratinocyte SFM:DMEM/F12 (1:1) medium, containing 5% FBS, and cultured for 1 day. The next day, the cells were incubated with adipogenic or osteogenic differentiation medium. For adipogenic differentiation, hUDSCs were incubated with DMEM, containing 10% FBS, 1% penicillin/streptomycin, 1 μM dexamethasone (Sigma-Aldrich), 500 μM 3-isobutyl-1-methylxanthine (IBMX; Sigma-Aldrich, MO, USA), 5 μg/ml insulin (Sigma-Aldrich) and 10 μM rosiglitazone (Cayman chemical, USA) for 24 days. The medium were changed every 3 days. After differentiation, cells were fixed using 4% paraformaldehyde and stained with Oil-red-O (Sigma-Aldrich). For osteogenic differentiation, one day after seeding, hUDSCs (1.5×10^5^ cells/well in a 6-well plate) were incubated in DMEM, containing 10% FBS, 1% penicillin/streptomycin, 0.1 μM dexamethasone, 50 μM ascorbate-2-phosphate (Sigma-Aldrich), and 10 mM β-glycerophosphate (Sigma-Aldrich). Medium was changed every 3 days. On day 24, cells were fixed using 4% paraformaldehyde and stained with Alizarin Red S (Sigma-Aldrich).

### Differentiation into IPCs

To induce differentiation into IPCs, hUDSCs were seeded (2.5×10^5^ cells/well) in DMEM/F12 (ThermoFisher Scientific), containing 1% B27 (ThermoFisher Scientific), 1% N2 (ThermoFisher Scientific), 50 ng/ml activin A (Peprotech, USA), 2 mM valproic acid (VPA; Sigma-Aldrich), and 1% penicillin/streptomycin, in a low-attach 12-well plate (SPL Life Science, Korea) for the first 3 days. On day 4, the medium was changed to DMEM/F12, containing 1% B27, 1% N2, 10 nM exendin-4 (Sigma-Aldrich), 10 ng/ml basic fibroblast growth factor (bFGF; Sigma-Aldrich), and 1% penicillin/streptomycin. On day 7, the medium was changed to DMEM/F12, containing 1% B27, 1% N2, 10 nM exendin-4, 50 ng/ml hepatocyte growth factor (HGF; Peprotech), 10 mM nicotinamide (Sigma-Aldrich), and 1% penicillin/streptomycin, which was changed every 3 days, up to 15 days.

### Glucose-stimulated insulin secretion assay

After 15 days of IPC differentiation, cells were washed twice with PBS. The cell clusters were incubated with 0.2% glucose Krebs (KRB) buffer for 1 h at 37°C to synchronize. Thereafter, the clusters were incubated with KRB buffer, containing 3 or 20 mM glucose for 1 h at 37°C. The supernatants were collected and diluted 10-fold, and released insulin was measured using an enzyme-linked immunosorbent assay (ELISA) kit, according to the manufacturer's protocol (ALPCO, USA). Insulin content was normalized to protein levels, which was determined using a Pierce BCA Protein Assay kit (ThermoFisher Scientific).

### Quantitative real-time polymerase chain reaction (qRT-PCR)

qRT-PCR was followed the methods of Cha *et al*.^15^, briefly, total RNA was extracted from the cells using RNAiso Plus (Takara Bio Inc., Japan) and cDNA was prepared using the PrimeScript™ cDNA synthesis kit (Takara Bio Inc., Japan) according to the manufacturer's instructions. cDNA samples were analyzed by the SYBR® Premix Ex Taq™, ROX plus (Takara Bio Inc.) on Bio-Rad cyclers (USA). Gene expression was normalized to the endogenous housekeeping control gene, cyclophillin. Relative expression was calculated for each gene using the ΔΔCT (where CT is the threshold cycle) method. The primer sequences used are listed in Table 1.

### Immunocytochemical staining

On day 15 of IPCs differentiation, clusters were washed with PBS and fixed using 4% paraformaldehyde in PBS at 4°C overnight. The fixed clusters were washed with PBS, then blocked with PBS, containing 1% BSA and 0.2% Triton X-100, for 1 h at room temperature, and incubated overnight with anti-Pdx1 (Millipore, Germany), anti-insulin (Abcam, UK), anti-glucagon (Cell signaling, USA), and anti-c-peptide (R&D systems, USA) at 4°C overnight. Clusters were then stained with a fluorescence-conjugated secondary antibody (Life Technologies, Carlsbad, CA) for 2 h at room temperature, the nucleus was stained with DAPI (5 μg/ml) for 10 min, mounted with VECTASHIELD (Vector Laboratories, USA), and observed under a confocal microscope (Zeiss, Germany). To evaluate PDX-1, insulin, glucagon and c-peptide, 8~15 clusters were imaged from three independent experiments. Image J program (https://imagej.nih.gov/ij/) was used to quantify the fluorescence intensity.

### Statistical analysis

Statistical analysis was carried out by one-way ANOVA with Bonferroni analysis. Data are presented as means ± SEM. Values of *p*<0.05 were considered significant.

## Results

### Isolation and characterization of hUDSCs

**Figure 1:**
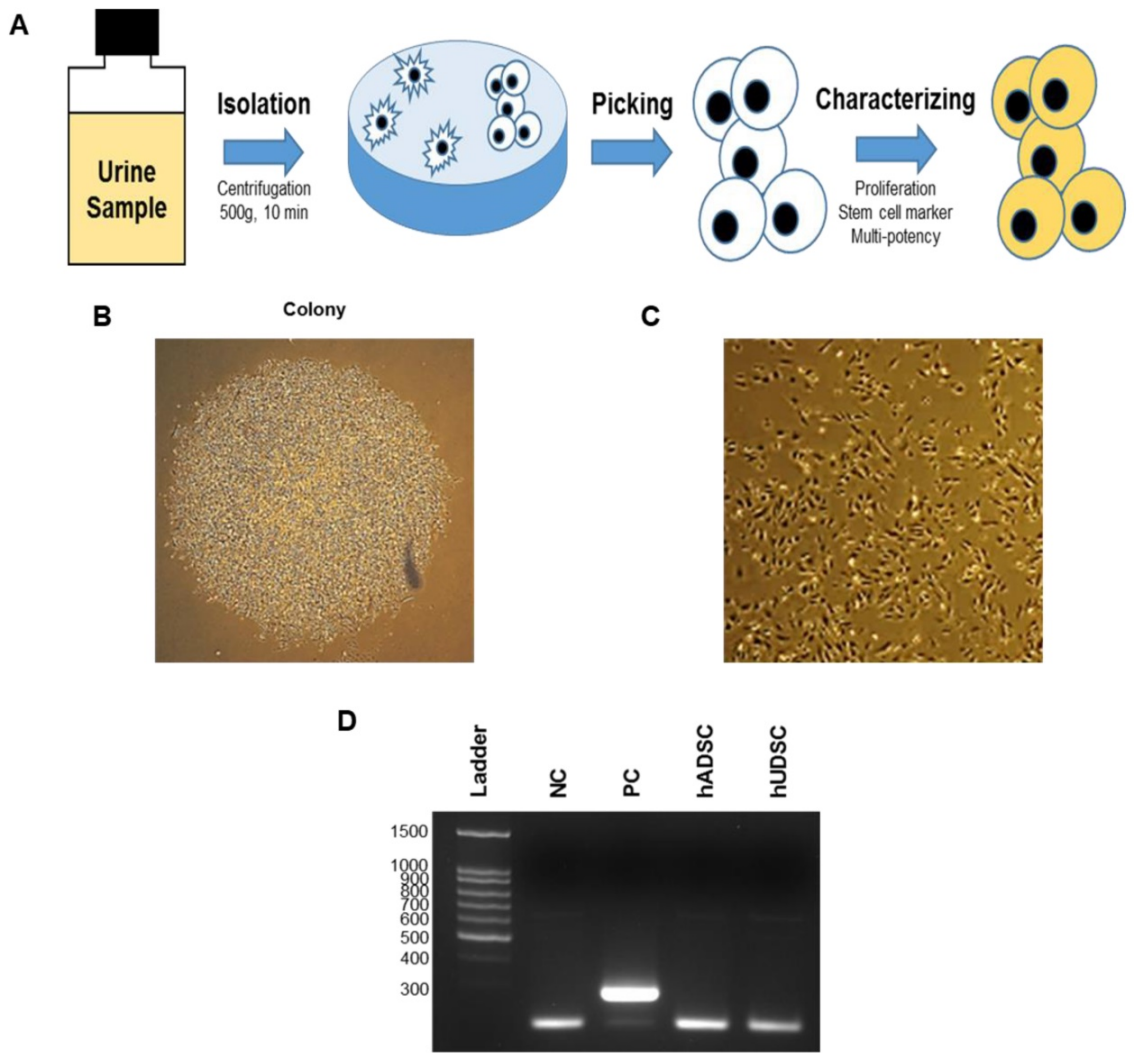
Isolation of hUDSCs. **(A)** Scheme of isolation of stem cells from human urine. **(B)** Colony formed at 5 days after plating. **(C)** Cell morphology. Magnification: 100×. **(D)** Mycoplasma test by RT-PCR. NC: negative control, PC: positive control. hUDSCs: human urine-derived stem cells, hADSCs: human adipose tissue-derived stem cells.

The hUDSCs isolation procedure is summarized in Figure 1A. The cells obtained from the urine samples were cultured and the adherent cells on the plate as a colony were transferred to a 6-well plate. Several different colonies were observed 5 days after initial seeding (Figure 1B). When the colony dissociated and was cultured for several passages, one of the selected clones, hUDSC001.01 showed an elongated morphology (Figure 1C). When we examined whether these cells were contaminated with mycoplasma by PCR, we confirmed that the cells were not contaminated with mycoplasma (Figure 1D).

**Figure 2:**
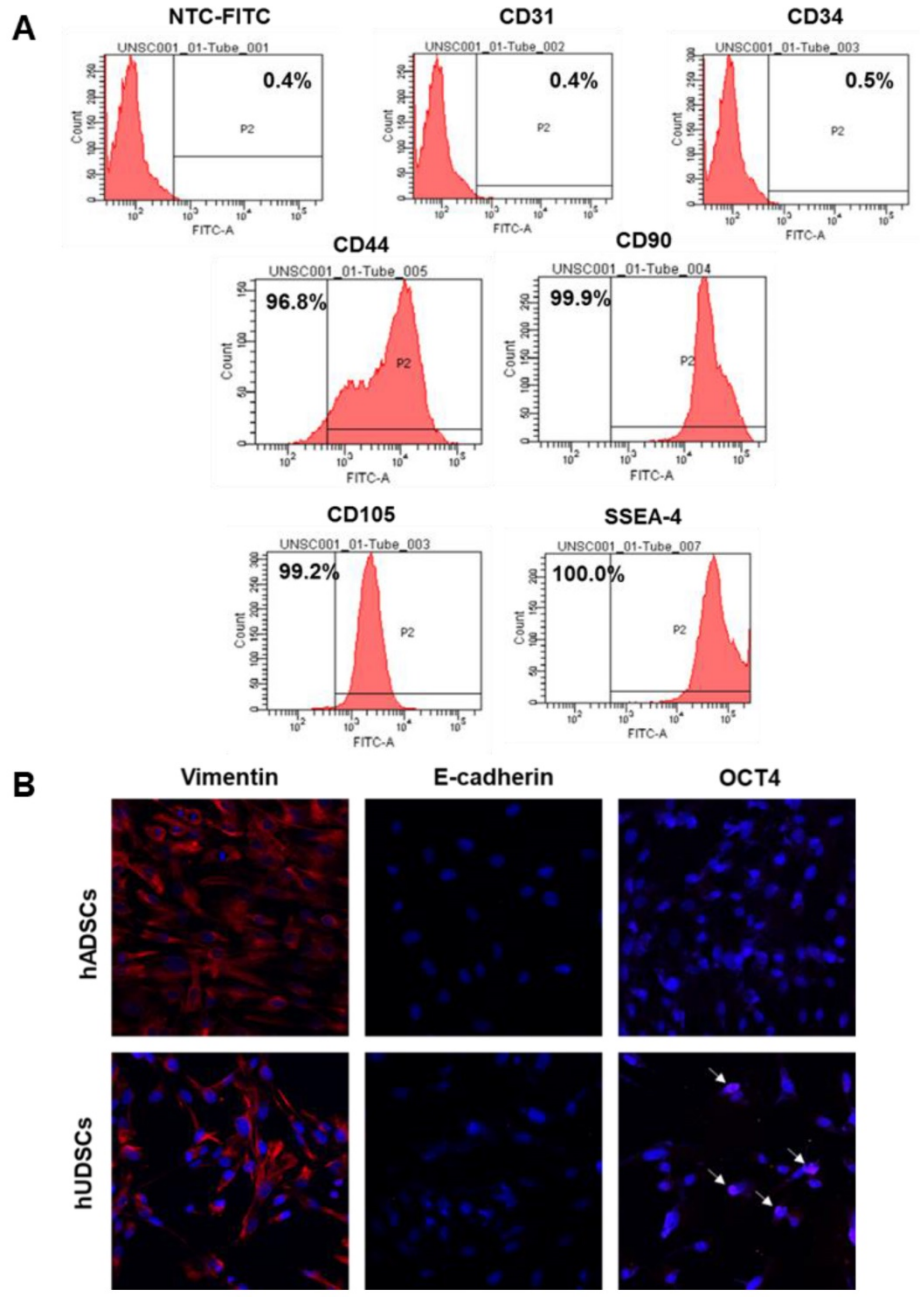
Characterization of hUDSCs for mesenchymal and stem cell markers. **(A)** The expression of CD31, CD34, CD44, CD90, CD105, and SSEA-4 was assessed by FACS analysis. **(B)** The expression of E-cadherin and OCT4 was analyzed by immunocytochemistry. hADSCs were used as a positive control. Magnification: 200×.

To examine the expression of stem cell surface markers in hUDSCs, we performed fluorescence-activated cell sorter (FACS) analysis after staining hUDSCs with antibodies against CD44, CD90, CD105, or SSEA-4. The cells obtained from urine were positive for mesenchymal stem cell antigens, such as CD44, CD90, and CD105. We found that more than 95% of the cells expressed CD44, and almost all the cells expressed CD90, and CD105, and SSEA-4, but not the endothelial (CD31, CD34) markers (Figure 2A). We next examined the protein expression of selected pluripotent markers in hUDSCs by immunocytochemistry. The protein expression for vimentin and Oct4 was detected, but E-cadherin was not expressed in hUDSCs. In contrast, human adipose-derived stem cells (hADSCs), which were used as a positive control, expressed vimentin only (Figure 2B).

### Differentiation of hUDSCs to adipocytes and osteocytes

**Figure 3:**
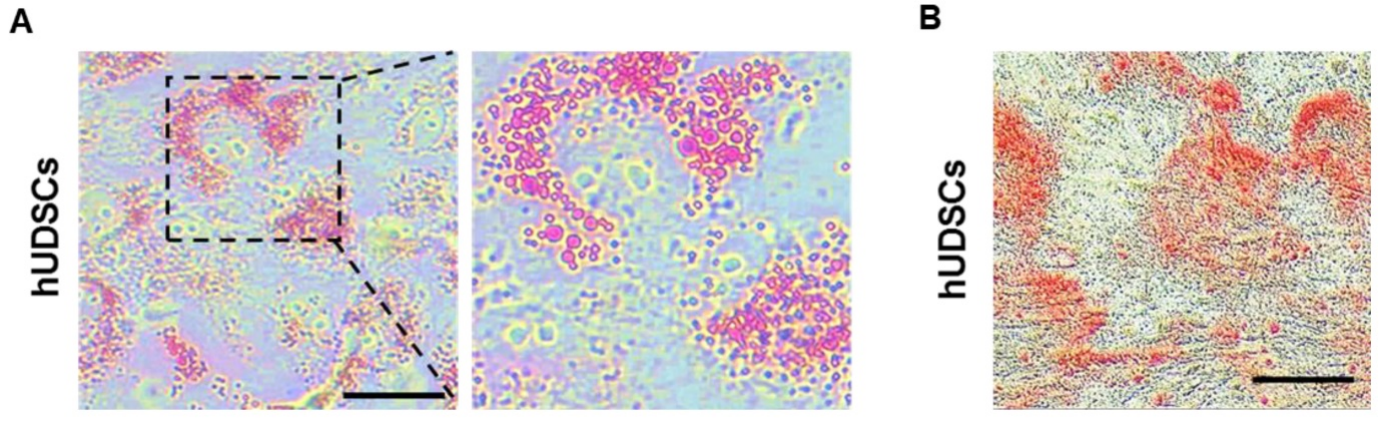
Differentiation of isolated hUDSCs into adipocytes and osteocytes. **(A)** hUDSCs were differentiated to adipocytes and stained by Oil-red-O. **(B)** hUDSCs were differentiated to osteocytes and stained by alizarin red S. Scale bars 20 µm.

Stem cells are able to proliferate indefinitely and can differentiate into various types of cells 16. Therefore, we examined whether the isolated hUDSCs can differentiate into adipocytes and osteocytes. The differentiated adipocytes showed Oil-red-O-stained cells (Figure 3A), suggesting that hUDSCs can differentiate into adipocytes. In addition, osteogenic differentiation was confirmed by Alizarin-positive cells, suggesting that the isolated hUDSCs can differentiate into osteocytes (Figure 3B).

### Differentiation of hUDSCs into IPCs

**Figure 4:**
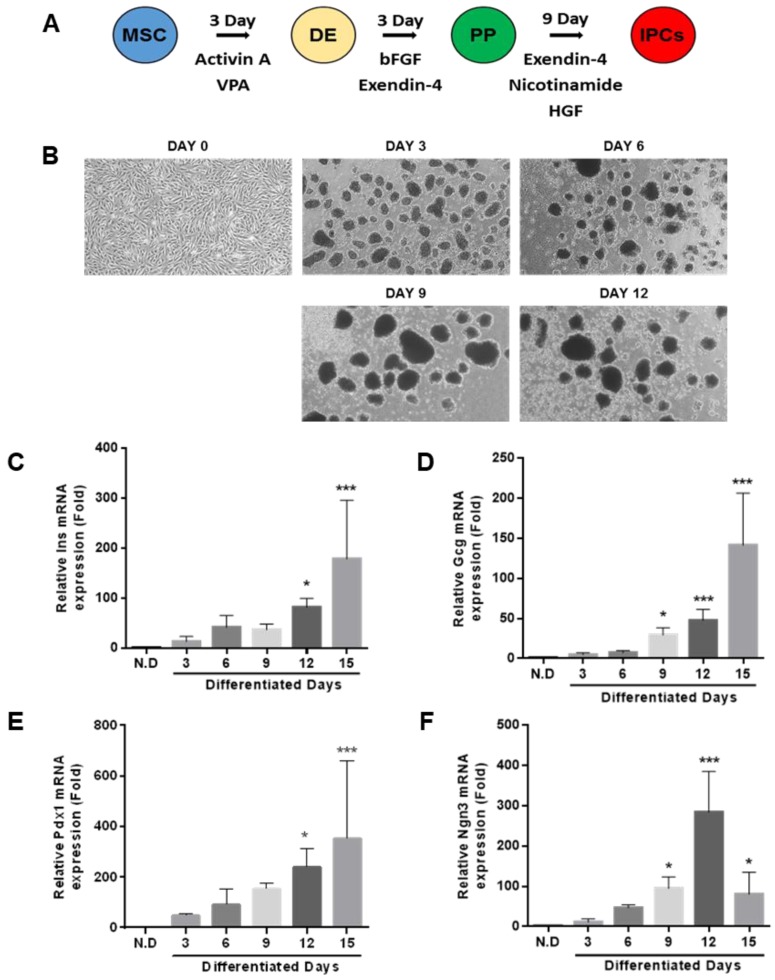
Differentiation of hUDSCs into IPCs. **(A)** Scheme for the procedure of hUDSC differentiation into IPCs. **(B)** Morphological changes during differentiation into IPCs. mRNA expression analysis of **(C)** Ins (insulin), **(D)** Gcg (glucagon), **(E)** Pdx1, and **(F)** Ngn3 was performed by RT-qPCR. N.D: non-differentiation. n=3-4, *p<0.05, ***p<0.005.

To investigate whether the isolated hUDSCs can directly differentiate into IPCs, we differentiated hUDSC to IPCs by a three-step differentiation protocol described in Figure 4A. Within a day, hUDSCs began to form clusters, which became more stable and compact as the differentiation progressed. The morphology of the differentiated IPCs showed smooth and round clusters (Figure 4B). We analyzed mRNA expressions of pancreatic transcription factors, such as pancreatic Pdx1 and Ngn3, and endocrine cell markers (insulin and glucagon) by RT-qPCR. mRNA expression of endocrine hormones, insulin and glucagon, was increased as differentiation progressed, whereas expression in undifferentiated hUDSCs was not detected (Figure 4C, D). mRNA expression of Pdx1 was increased as differentiation progressed (Figure 4E), and Ngn3 mRNA expression increased until day 12 after differentiation, but, the mRNA expression was decreased thereafter (Figure 4F).

### Glucose-responsive insulin secretion in IPCs differentiated from hUDSCs

**Figure 5:**
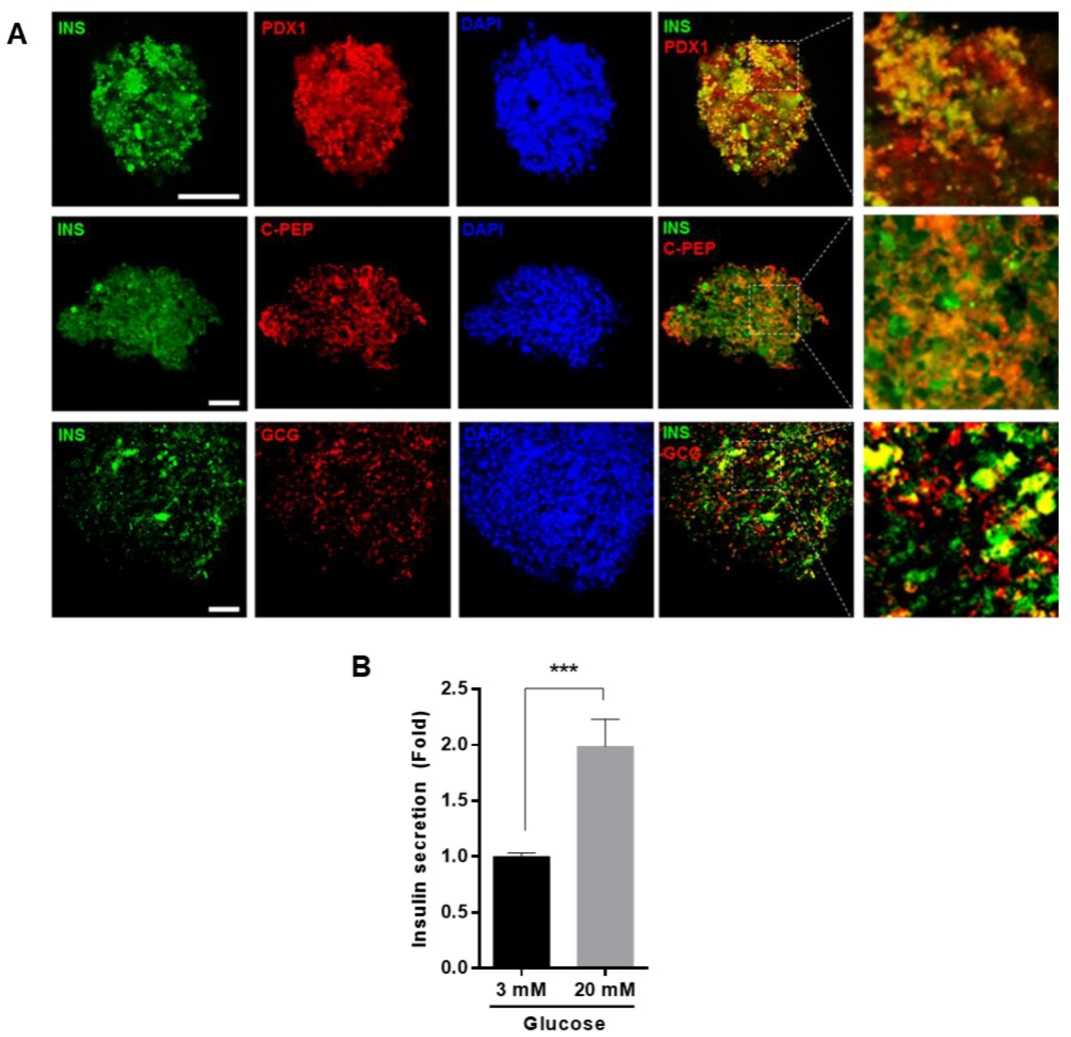
Differentiation IPCs from hUDSCs secreted insulin in response to glucose levels. **(A)** Differentiated IPC clusters were fixed and stained with anti-INS (green), anti-PDX1 (red), anti-C-PEP (peptide) (Red) or anti-GCG (red) antibodies and observed by confocal microscopy. Blue indicates DAPI (nuclear) staining. Scale bar indicates 50 µm. **(B)** Insulin secretion from differentiated IPCs in the presence of 3 mM and 20 mM glucose was analyzed by as described in “Materials and Methods”. n=3, ***p<0.005.

To confirm that the differentiated cells expressed insulin and glucagon proteins, we performed immunocytochemistry, using anti-insulin, anti-Pdx1, anti-C-peptide, and anti-glucagon antibodies. Differentiated cells were positive for insulin and glucagon. In addition, Pdx1 and C-peptide, a cleavage product of insulin, were co-localized with insulin, suggesting that the differentiated cells clearly produced insulin (Figure 5A). To investigate whether differentiated IPCs could increase insulin secretion in response to glucose, the IPCs were cultured in the presence of low (3 mM) or high glucose (20 mM) concentration and the amount of secreted insulin was measured. We found that insulin secretion was increased about 2-fold in the presence of high glucose compared with that in the presence of low glucose concentration (Figure 5B).

## Discussion

Type 1 diabetes results from absolute insulin deficiency due to autoimmune-mediated destruction of pancreatic β-cells 17. Therefore, daily insulin injections are required for the survival for type 1 diabetic patients. However, exogenous insulin injections cannot tightly regulate insulin levels as endogenous β-cells do in response to physiological changes in blood glucose levels. Therefore, islet transplantation is considered to be an appropriate therapeutic strategy for the treatment of type 1 diabetes; however, the lack of islet donors is a limitation for this therapy. As an alternative source to solve this limitation, differentiated islet-like cells from stem cells, including mesenchymal stem cells, embryonic stem cells, and induced pluripotent stem cells, have been investigated 18, 19.

Mesenchymal stem cells are found in a number of tissues, and may have the ability to trans-differentiate into multiple cell types including adipocytes, osteocytes, myocytes neuronal cells, and islets-like cells 20. Therefore, the mesenchymal stem cells can be obtained from adipose tissue, bone marrow, muscle, and so on. However, the isolation of stem cells from most tissues requires invasive procedures 21-26.

Stem cell isolation from urine is non-invasive, simple, and easy 10, 11, 27. In this study, we isolated mesenchymal stem cells from human urine, and characterized and investigated their differentiation potential to IPCs. The isolated hUDSCs expressed CD44, CD90, CD105, and SSEA-4. Cells expressing SSEA-4, an embryonic stem cells marker, have proliferation ability and multi-potency 28. In addition, it is known that mesenchymal stem cells express CD105, CD90, and CD44, but not CD34, CD31, and CD45 29. These results indicate that stem cells isolated from urine may have a mesenchymal stem cell characteristic and multi-potency.

Several stem cell markers, Oct4, sex determining region Y-box2 (Sox)2, Rex1, and Nanog, are needed to maintain self-renewal and the undifferentiated state of stem cells 30-33. Oct4 was found in oocytes, mouse embryos and germ cells, as well in embryonic stem cells, but not in the differentiated cells 34. Vimentin is also a kind of stem cell marker, particularly expressed in mesenchymal stem cells. The expression of Oct4 and vimentin was also detected in hUDSCs in our study, whereas the Oct4 was not detected in hADSCs. These results suggest that hUDSCs display pluripotent stem cell markers and might have differentiation potential.

It was reported that stem cells from urine can differentiate into osteoblast, adipocyte, or skeletal myocyte lineages *in vitro* 12, 22, 27. Therefore, we also confirmed that the isolated hUDSCs can differentiate into osteocytes and adipocytes. Direct differentiation of mesenchymal stem cells obtained from different sources into IPCs have been reported 25, 29, 35. However, it is not known whether hUDSCs can be directly differentiated into IPCs. So, we investigated whether hUDSCs have a potential to directly differentiate into IPCs. When we differentiated hUDSCs to IPCs using combinations of defined factors for β-cell differentiation as described previously 35, the expression of mRNA and proteins for PDX1 (a pancreatic lineage specific marker) and NGN3 (an endocrine progenitor marker) was observed during the differentiation. In addition, mRNAs and proteins for insulin and glucagon were also detected. These results indicate that isolated hUDSCs have the potential to directly differentiate into IPCs. The most important characteristic for IPCs is the secretion of insulin in response to increase in glucose. Therefore, we examined whether the differentiated IPCs could secrete insulin in response to glucose. We found that the differentiated IPCs increased insulin secretion in the presence of high glucose concentration, compared with low glucose, suggesting that these differentiated IPCs are functional, although the degree of response to glucose was less than the primary islets.

## Conclusion

We demonstrated that the isolated hUDSCs expressed the mesenchymal stem cell markers and can be directly differentiated into IPCs. The differentiated IPCs expressed pancreatic transcription factors, insulin and glucagon. In addition, these IPCs secrete insulin in response to glucose. Although further investigations are needed, our results suggest that hUDSCs might be useful source for cell therapy in type 1 diabetes.

## Figures and Tables

**Table 1:** RT-PCR primer sequences

Gene name		Sequence 5' - 3'
Pdx1	Forward	GAA CTT GAC CGA GAG ACA CAT CAA
	Reverse	TTG TCC TCC TCC TTT TTC CAC TT
Ngn3	Forward	CGG AGT CGG CGA AAG AAG
	Reverse	CGT CCA GTG CCG AGT TGA G
Insulin	Forward	GCA GCC TTT GTG AAC CAA CA
	Reverse	TTC CCC GCA CAC TAG GTA GAG A
Glucagon	Forward	CCC AAG ATT TTG TGC AGT GGT T
	Reverse	CAG CAT GTC TCT CAA ATT CAT CGT
Cyclophillin B	Forward	TGC CAT CGC CAA GGA GTA G
	Reverse	TGC ACA GAC GGT CAC TCA AA
